# Damage law and mechanism of coal-rock joint structure induced by liquid nitrogen at low temperature

**DOI:** 10.1038/s41598-022-15185-8

**Published:** 2022-06-28

**Authors:** Hewan Li, Siyang Sun, Laigui Wang, Jian Liu, Ziheng Zhang

**Affiliations:** College of Mechanics Engineering, Liaoning Technical University, No. 47, Zhonghua Road, Xihe District, Fuxin, 123000 Liaoning China

**Keywords:** Environmental sciences, Environmental social sciences, Energy science and technology, Engineering, Materials science

## Abstract

The width and degree of connectivity of coal-rock joints directly affect the seepage capacity of flow energy such as gas. To study the damage law and mechanism of the coal-rock joint structure under the action of liquid nitrogen, two methods of liquid nitrogen unloaded and liquid nitrogen freeze–thaw were used to carry out damage modification experiments on coal-rock with different water saturation. Using OLS4000 laser confocal microscope and MH-25 universal testing machine to conduct electron microscope scanning and uniaxial compression test, measure the joint width expansions and Young's modulus of the coal-rock surface before and after the test, establish a physical and mechanical model of freeze–thaw damage to analyze the ice-wedge expansion stress influence on the damage of coal-rock joint structure and establish damage criterion. The research results show that the ice-wedge expansion stress, confining pressure, and temperature stress in the joint jointly affect the structural damage of coal-rock joints, and the ice-wedge expansion stress contributes the most. With the increase of water saturation, the damage to the coal-rock joint structure intensifies, and the ice-wedge expansion stress under the water saturation state has the most obvious influence on the damage to the coal-rock joint structure. The damage criterion constructed by the freeze–thaw damage physical–mechanical model can reveal the damage mechanism of the effect of ice-wedge expansion stress on the coal-rock joint structure. This paper has certain practical significance for the safety and stability evaluation of rock engineering in cold and arid regions and provides new ideas for effectively extracting clean energy such as coalbed methane and preventing rock bursts.

## Introduction

Increasing global energy demand is a driving factor for the production of natural gas resources^1^. Due to the special geological structure and burial conditions of coalbed methane in China, coal reservoirs have the characteristics of low porosity, low permeability, and low reservoir pressure, which seriously hinder the extraction of coalbed methane^2,3^. With the increase of the mining depth, the gas content and reservoir pressure in the coalbed methane also gradually increase, which makes the disasters such as gas explosions and gas outbursts become more and more common and serious^4,5^. In particular, the production of coalbed methane in severe cold regions and arid regions of China is mainly limited to coal reservoirs with high gas content, large burial depth, and low permeability. Therefore, understanding the structural characteristics of coalbed methane and effectively extracting coalbed methane is of great significance to alleviate the problem of resource shortage in China^6^, optimizing the energy structure, and preventing environmental pollution.

Since the twentieth century, various experimental methods have been used to study the damage process of coal rock, including micromechanical methods^7–11^, acoustic emission technology^12,13^, CT scanning technology^14^, and so on. Using micro-mechanical methods, a crack model is established to study the growth, development, and aggregation of micro-cracks, and the influence of micro-cracks on mechanical properties is reflected in the constitutive relationship. However, the interaction between cracks is neglected, and the damage model is mainly limited to the pre-peak nonlinear hardening state^7^. Using acoustic emission techniques to explore fracture-related acoustic emission characteristics in defective rocks, mainly focusing on intact rocks. However, natural rocks usually contain many pre-existing defects, such as joints, faults, and fractures, making the failure mechanisms of defective rocks significantly different from those of intact rocks. The relationship between the unstable failure stage, acoustic emission event rate, and load increase (instantaneous strain energy release) of defective rocks is currently unclear^13^. CT scanning technology is used to observe the distribution of cracks in the internal cross-section of rocks under a certain stress state. However, CT scanning technology can only scan damaged rock samples at a given stage in the past, and cannot obtain real-time CT images of different stress stages in the evolution of rock damage^14^. Temperature also has a certain influence on the damage of coal rock. Under the influence of temperature, the strength of coal rock will decrease significantly until it is destroyed. Low-temperature freezing of coal- rock is a current global scientific problem, which has certain practical significance for the safety and stability of rock engineering construction in severe cold regions around the world. Therefore, it is necessary to study the damage law and mechanism of coal-rock joint structure induced by liquid nitrogen at low temperatures.

Liquid nitrogen is an environmentally friendly ultra-low temperature fluid, and the temperature under normal temperature and pressure (0.1 MPa) is − 196 °C. The injection of liquid nitrogen into the coal reservoir will greatly reduce the temperature of the reservoir, causing a temperature gradient between the inner and outer coal and rock, resulting in tensile stress on the surface of the coral rock. When the tensile stress exceeds the ultimate tensile strength, cracks will occur on the surface of the coral rock. At 21 °C, 1m^3^ of liquid nitrogen is vaporized to 696 m^3^ of nitrogen, and the latent heat of vaporization is 5.56 kJ/mol. During the evaporation of liquid nitrogen, the water in the coal-rock fractures will freeze rapidly, expand by 9% in volume, and accumulate at the tip of the coal-rock fracture to generate a frost heave force of 210 MPa^15^, which promotes the expansion of the original fractures and the expansion of new fractures.

In the process of exploring the improvement of coalbed methane extraction, it was found that traditional techniques such as hydraulic fracturing and hydraulic grooving have the limitations of high water consumption and are difficult to implement in arid areas. Chemical methods such as acid treatment^16^ and solvent extraction have the disadvantages of polluting the water environment and blocking the seepage channel with fracturing fluid. To improve the permeability of coal reservoirs in China, researchers tried to inject low-temperature fluids such as liquid nitrogen into coal reservoirs to increase the production of coalbed methane, and this fracturing method was successfully tested in the San Juan Coalbed Gas Field in New Mexico, USA. Compared with the traditional fracturing method, the gas production rate is increased by 8%, indicating that liquid nitrogen can effectively destroy the original reservoir structure and improve the permeability of the reservoir coal-rock. In recent years, scholars have studied the fracture evolution, mechanical properties, and permeability changes of coal-rock after liquid nitrogen treatment. Zhai et al.^17^ studied the change in the fissure structure of coal samples after liquid nitrogen treatment and found that the liquid nitrogen freeze–thaw cycle destroyed the fissure structure of the coal-rock, and at the same time increased the fissure density and the overall permeability of the coal-rock. Qin et al.^18^ studied the effect of liquid nitrogen freeze–thaw on the mechanical properties of coal-rock and found that compared with the freezing time, the freeze–thaw cycle has a greater damage intensity to the coal-rock. In the study of permeability characteristics, Yin^19^ et al. found that with the increase of water content of coal samples, permeability increased and fractures developed rapidly, indicating that the effect of low temperature freezing and thawing on tighter coal rocks is more significant. Wei et al.^20^ studied the change law of coal permeability under cold shock and hot and cold shock conditions and found that after cold shock and hot and cold shock, the average increase rate of coal permeability was 48.68% and 469.24%, respectively. It can be seen that the permeability of coal rock after liquid nitrogen treatment is improved, which is of great significance to the efficient exploitation of coalbed methane.

Although liquid nitrogen low-temperature fracturing has broad application prospects, it is not clear how coal-rock joints change in the process of liquid nitrogen low-temperature freezing, and whether liquid nitrogen low-temperature fracturing can effectively destroy the coal-rock joint structure. This paper mainly solves the following three problems: (1) How is the surface joint structure of coal samples damaged under the action of liquid nitrogen unloaded and liquid nitrogen freezing and thawing; (2) How to deduce the freezing and thawing damage solution formula and obtain the damage criterion; (3) according to the test results and theoretical model, analyze what are the key factors leading to the damage of the coal-rock joint structure. This study can provide a reference for the safety and stability evaluation of rock engineering in cold and arid regions.

## Materials and methods

### Coal sample preparation

The test coal samples were collected from long flame coal from Ping'an Mine (121° 67′ E, 42° 02′ N) in Fuxin City, Liaoning Province, China. To ensure the uniformity of the test coal samples, all coal samples were drilled in the same coal block along the vertical bedding direction and immediately sealed with plastic wrap to prevent pollution and oxidation. In the laboratory, the drilled coal samples were ground and processed into cubes with a flat surface of 100 mm × 100 mm × 100 mm. Before the test, a non-metallic ultrasonic detector was used to measure the longitudinal wave velocity of the coal sample, and the complete coal sample with the longitudinal wave velocity in the range of 1600–1660 m/s was selected to reduce the error caused by the dispersion of the coal sample on the test results^21^**.** The basic properties and labels of the coal samples are shown in Tables 1 and 2. The vertical bedding direction is z, and the parallel bedding directions are x and y.

**Table 1:** Basic properties of coal samples.

Coal sample name	Buried depth (m)	Approximate analysis (wt%)			
		*M*_ad_	*A*_ad_	*V*_daf_	*FC*_ad_
long flame coal	500 m	6.04	30.29	24.03	40.91

*M*_*ad*_ moisture content after air-drying, *A*_*ad*_ ash content after air-drying, *V*_*daf*_ volatile matter content after air-drying, *FC*_*ad*_ fixed carbon content after air-drying^22^.

**Table 2:** Coal sample labeling.

Coal sample classification	Water saturation 0%		Water saturation 50%		Water saturation 100%	
	Dry coal sample	Nitrogen	Saturation treatment	Nitrogen	Saturation treatment	Nitrogen
Label	P_5_S_0_L_0_	P_5_S_0_L_1_	P_5_S_50_L_0_	P_5_S_50_L_1_	P_5_S_100_L_0_	P_5_S_100_L_1_

### Experiment steps

**Figure 1:**
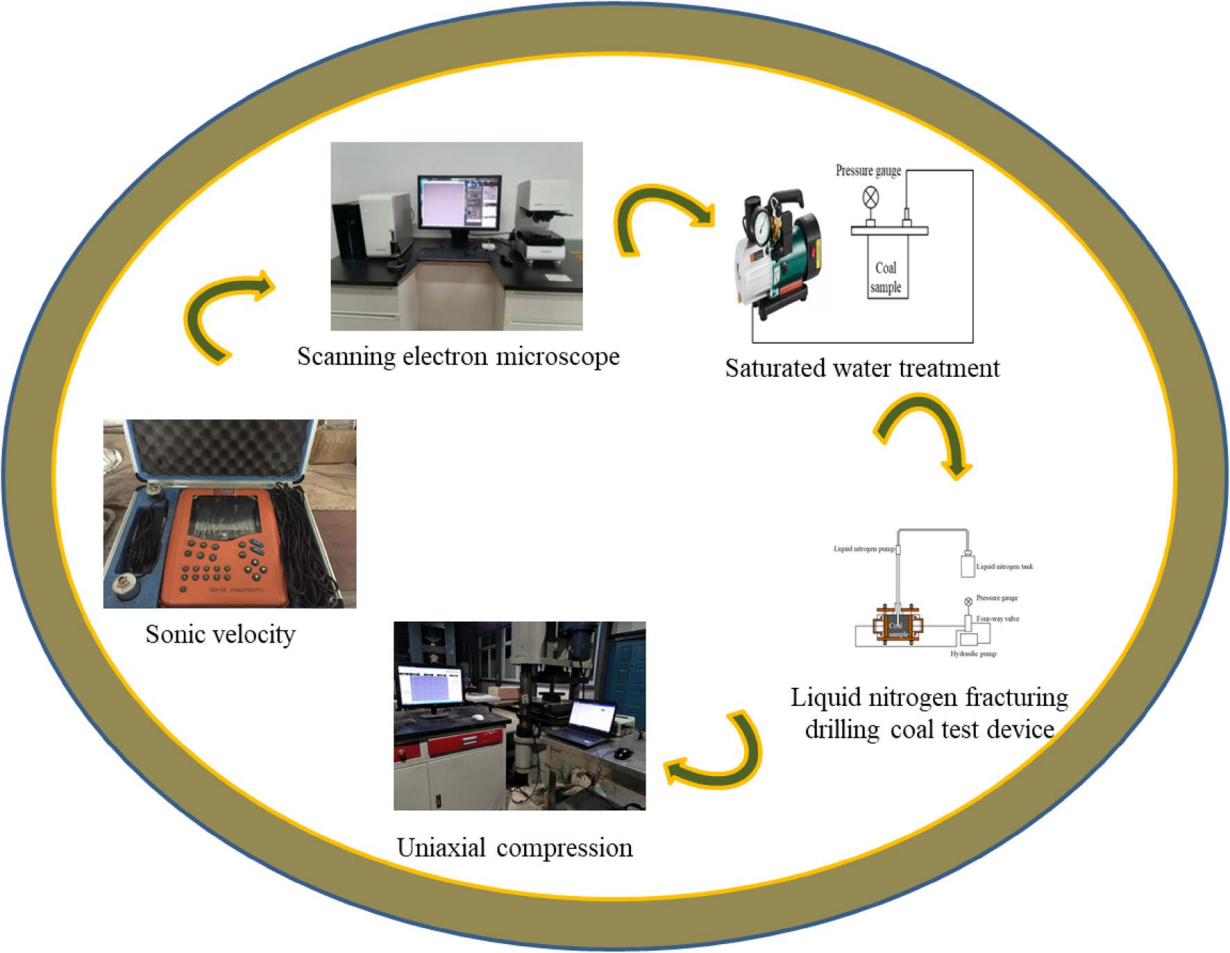
Test flow chart.

**Figure 2:**
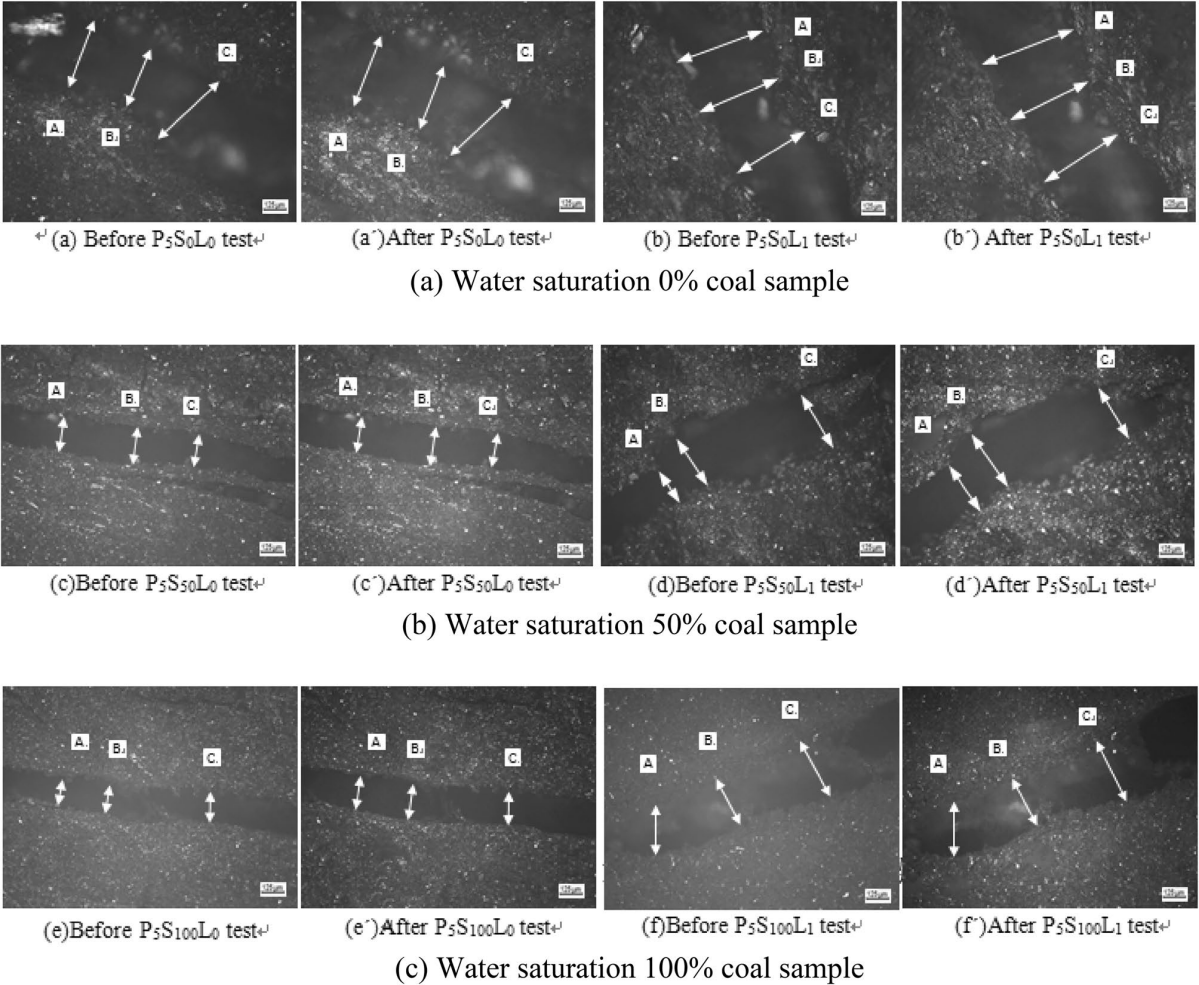
Comparison of coal sample joints before and after the test.

In this paper, a single-cycle comparative test is used for research. The test process is shown in Fig. 1. The test steps are as follows:

1. Use a non-metallic ultrasonic detector to measure sonic velocity and screen coal samples for testing;
2. Through visual inspection, a 5 mm observation area was delineated at the obvious joint of the coal sample, and the OLS4000 laser confocal microscope was used for electron microscope scanning to observe the mirror image of the coal sample surface magnified 100 times before and after the test. Select three fixed observation points A, B, and C on the joint image, as shown in Fig. 2, and calculate the joint expansion amount and expansion rate on the coal sample surface according to the scale on the figure.

The joint width extension $\Delta d$ on the coal sample surface can be expressed as

1 $$ \Delta d = d_{1} - d_{0} $$

In Eq. (1), $\Delta d$ is the expansion of the surface joint width of the coal sample before and after the test, μm; $d_{0}$ is the joint width before the test, μm; $d_{1}$ is the joint width after one cycle of the test, μm.

The joint width expansion rate $\updelta$ on the surface of the coal sample is calculated by using the expansion amount of the surface joint width of the coal sample before and after immersion in liquid nitrogen, that is,

2 $$ \delta = \frac{{d_{1} - d_{0} }}{{d_{0} }} \times 100\% $$

(3) Treatment of coal samples with different water saturation by vacuum pump.

Put the water and coal samples into the vacuum stainless steel tank, and use the air pump to evacuate the steel tank to discharge the air from the pores and fissures of the coal sample, weighing once every 0.5 h. When the quality of the coal sample no longer increases, it is considered that the coal sample is forced to be saturated with water. The water saturation of the coal sample is defined according to the water saturation time and the weight gain percentage, and the water saturation of 0%, the water saturation of 50%, and the water saturation of 100% are obtained respectively coal sample;

(4) Comparative test.

**Figure 3:**
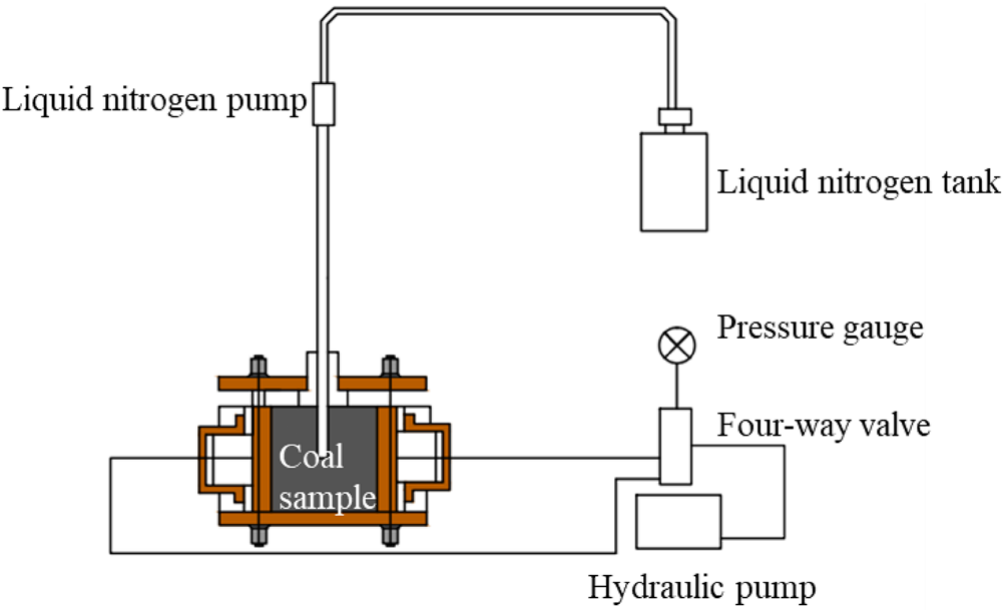
Liquid nitrogen fracturing drilling coal test device.

***A*** Liquid nitrogen no-load test: The liquid nitrogen fracturing drilling coal test device was used to apply only 5 MPa confining pressure to the coal sample, and the loading was 24 h as a cycle, and the liquid nitrogen freeze–thaw treatment was not performed on the water-containing coal sample. The test device is shown in Fig. 3;

***B*** Liquid nitrogen freeze–thaw test: A confining pressure of 5 MPa was applied to the coal sample by using the liquid nitrogen cracking drilling coal test device. At the same time, the water-containing coal sample was subjected to liquid nitrogen freeze–thaw treatment, injected with liquid nitrogen for 4 h, and placed at room temperature (25 °C) for 20 h as a freeze–thaw cycle.

**Figure 4:**
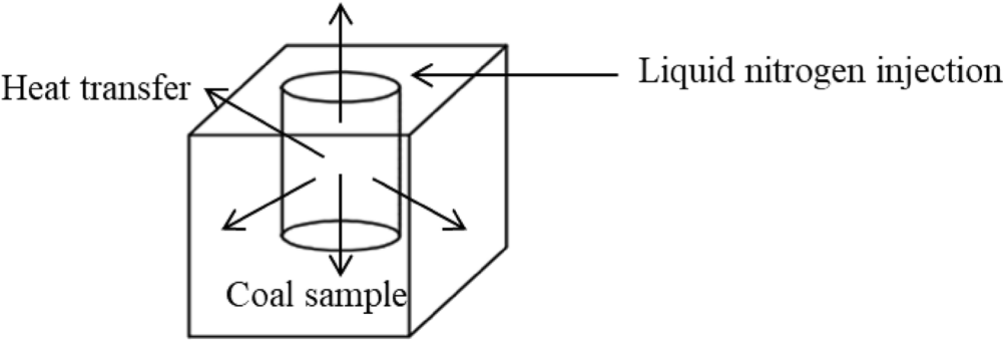
Schematic diagram of heat transfer.

When the coal sample is injected with liquid nitrogen, the liquid phase water in the joint turns into solid ice in a relatively short period, and the volume expands by about 9%^23^, forming an ice wedge expansion force in the joints and fissures of the coal sample. When the ice-wedge expansion stress is greater than the tensile strength limit of the coal sample (0.52 MPa), the coal sample is squeezed outward and damaged, or even split. When the solid phase ice heats up and turns into liquid-phase water, the structural deformation of the coal sample cannot be completely recovered, resulting in permanent plastic deformation. Under the combined action of ice-wedge expansion stress, temperature stress, and confining pressure, the volume expansion and deformation of the coal sample are limited, and the inner side of the borehole is first deformed and damaged in the inward normal direction by the confining pressure. The heat transfer is shown in Fig. 4.

(5) Based on (4), the MH-25 universal testing machine was used to carry out a uniaxial compression test of coal samples for ***A*** and ***B*** respectively, and the stress–strain relationship curve and compressive strength were obtained, revealing that the damage law of coal-rock joint structure with different water saturation under liquid nitrogen unloaded and liquid nitrogen freeze–thaw conditions ;

(6) A physical and mechanical model of freeze–thaw damage was established to analyze the damage mechanism of the coal sample joint structure.

According to the analysis of the damage to the joint structure on the surface of the coal sample and the uniaxial compressive strength, it is found that the liquid water in the joint of the coal sample freezes into solid ice, and the resulting ice-wedge expansion stress squeezes the joint wall. Under the action of liquid nitrogen freezing and thawing, the theoretical expansion stress generated when 1 g of water freezes under completely closed conditions is about 696 MPa^24^, which is far greater than the tensile strength of the coal sample joint structure of 0.52 MPa. When the coal sample joint structure is affected by the temperature stress $\upsigma _{t}$, the ice-wedge expansion stress $P_{f}$ and the confining pressure $\upsigma _{3}$, the ice-wedge expansion stress plays a decisive role. Therefore, when studying the freeze–thaw damage of coal sample joint structure, the ice-wedge expansion stress is the key to the research of the damage mechanism.

## Results and discussion

### Variation of joint structure on the coal sample surface

Under the action of a confining pressure of 5 MPa, two groups of tests were carried out for coal samples with a water saturation of 0%, 50%, and 100%, liquid nitrogen unloaded and liquid nitrogen freezing and thawing, respectively. The joints whose included angle with the bedding is greater than 45° but less than 90° are selected as the research objects, and the expansion effect of the joints on the surface of the coal sample before and after the test is observed at 100 times magnification, as shown in Fig. 2a–c shown. Measure the joint width at fixed positions (A, B, C) on the surface of the coal sample, and calculate the joint expansion amount and joint expansion rate on the surface of the coal sample, as shown in Table 3.

**Table 3:** Surface joint expansion amount and joint expansion rate of coal samples with different saturation degrees.

Coal sample numbering	*d*_0_/μm				*d*_1_/μm				Expand, *Δd*/μm	Expand rate, *δ*
	A	B	C	Average	A	B	C	Average		
P_5_S_0_L_0_	316.17	248.67	377.83	314.22	340.52	294.24	401.91	345.56	31.34	0.09974
P_5_S_50_L_0_	170.68	198.75	176.81	182.08	181.39	219.92	245.94	215.75	33.67	0.18492
P_5_S_100_L_0_	132.75	151.50	135.25	140.84	172.28	188.50	178.23	179.67	38.83	0.2757
P_5_S_0_L_1_	336.32	331.27	323.08	330.22	373.57	350.47	388.51	370.85	40.63	0.12304
P_5_S_50_L_1_	211.51	292.48	305.29	269.76	303.63	318.24	345.85	322.57	52.81	0.19577
P_5_S_100_L_1_	280.06	280.57	317.53	292.72	349.68	368.67	383.24	369.20	74.48	0.25444

**Figure 5:**
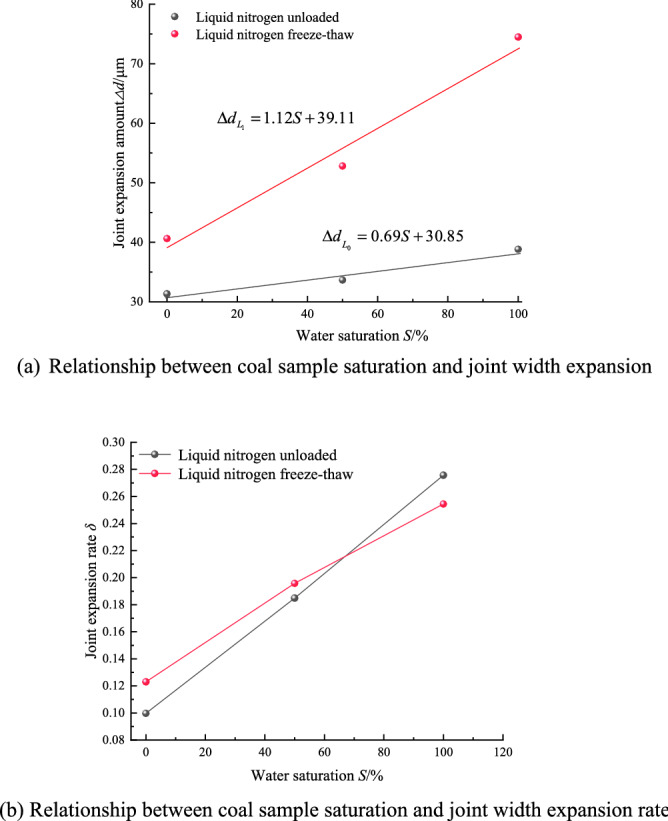
The curve of the test result of the change of the joint structure on the surface of the coal sample.

Fitting the coal sample saturation with the joint width expansion amount and the joint width expansion rate, the fitting curves are obtained as shown in Fig. 5a,b.

It can be seen from Fig. 5a that the joint expansion amount on the surface of the coal sample increases gradually with the increase of water saturation, no matter under the action of liquid nitrogen unloaded or under the action of liquid nitrogen freezing and thawing. According to the slope of the fitting curve, the freeze–thaw effect of liquid nitrogen is more obvious than that of liquid nitrogen without load, and the difference in growth rate between the two is 0.43. When the water saturation of the coal sample is 0%, the difference in the joint width expansion between the two is the smallest, which is 9.29 μm; when the water saturation of the coal sample is 100%, the difference in the joint width expansion between the two is the largest, which is 35.65 μm. It can be seen from Fig. 5b that with the increase of water saturation, the joint expansion rate of liquid nitrogen freeze–thaw increases more rapidly than that of liquid nitrogen unloaded, which shows that liquid nitrogen freeze–thaw has more severe damage to coal sample joints^25,26^. Only under the action of liquid nitrogen, the joint width of the dry coal sample was only affected by the temperature stress, and the joint width expanded by 5.32 μm; under the action of the confining pressure of 5 MPa, the width of the coal sample joint expanded significantly by 31.34 μm. It shows that the confining pressure has a great promoting effect on the damage of the joint structure of the dry coal sample, and it also shows that the temperature stress has a weaker effect on the damage of the joint structure of the coal sample than the confining pressure.

In observation and comparison test ***A***, it was found that with the increase of water saturation, the joint width of coal samples gradually increased, and the difference in joint width between coal samples with water saturation of 50% and coal samples with a water saturation of 0% was 2.33 μm. The difference in joint width between coal samples with water saturation of 100% and coal samples with water saturation of 0% is 7.49 μm. It can be seen from the analysis that this is because the liquid phase water plays a lubricating effect on the relative motion of the coal matrix particles. Under the action of confining pressure, the coal matrix particles accelerate and slip along the defect direction, resulting in increased damage to the joint structure. However, the joint structure expansion of coal samples with a water saturation of 100% is also affected by the liquid water pressure, so the joint width changes more obviously. At the same time, it also shows that the liquid-phase water in the joint has a weak effect on the damage of the coal sample structure when the phase transition does not occur.

Observing the comparison test ***B***, it was found that under the action of liquid nitrogen freezing and thawing, the volume of liquid-phase water became larger after being frozen into ice, which squeezed the joint wall. At this time, coal sample joints not only have the lubricating effect of liquid water on coal matrix particles before freezing but also the comprehensive effect of ice-wedge expansion stress, which causes the expansion of coal sample joints^27–30^. From the test results, the joint width expansion of the coal sample with a water saturation of 50% is 52.81 μm, while that of the coal sample with a water saturation of 100% is 74.48 μm. It can be seen that the higher the saturation, the more obvious the joint width expansion. The reason for the increase in the joint width of coal samples with a saturation of 50% is that there is a phenomenon where the saturation is greater than 91.7% in the local joints. When liquid nitrogen quickly freezes the liquid water into solid ice, it also causes local joint freezing swelling damage. Coal-rock is a brittle material, and local damage can cause a chain reaction of damage to low-temperature brittle materials, resulting in increased damage in a certain area^31–36^. Zhou et al.^14^ quantitatively analyzed the damage evolution process inside the rock through CT scanning technology, and proposed the damage variable equation. In this paper, the damage change of coal-rock joint structure is analyzed by scanning electron microscope, and the surface joint width expansion amount and coal sample surface joint width expansion rate before and after liquid nitrogen soaking are proposed. The experimental results of the two are basically consistent, which verifies the importance of the research results in this paper. From the perspective of the whole test system, the main reason for the aggravation of coal sample damage is the ice-wedge expansion stress generated by the freezing of liquid water into ice, resulting in aggravated damage to the coal sample joint structure. Therefore, it will be more practical to study the damage mechanism of coal sample joint structure under the action of liquid nitrogen at low temperatures.

### Strength damage analysis of coal samples

**Figure 6:**
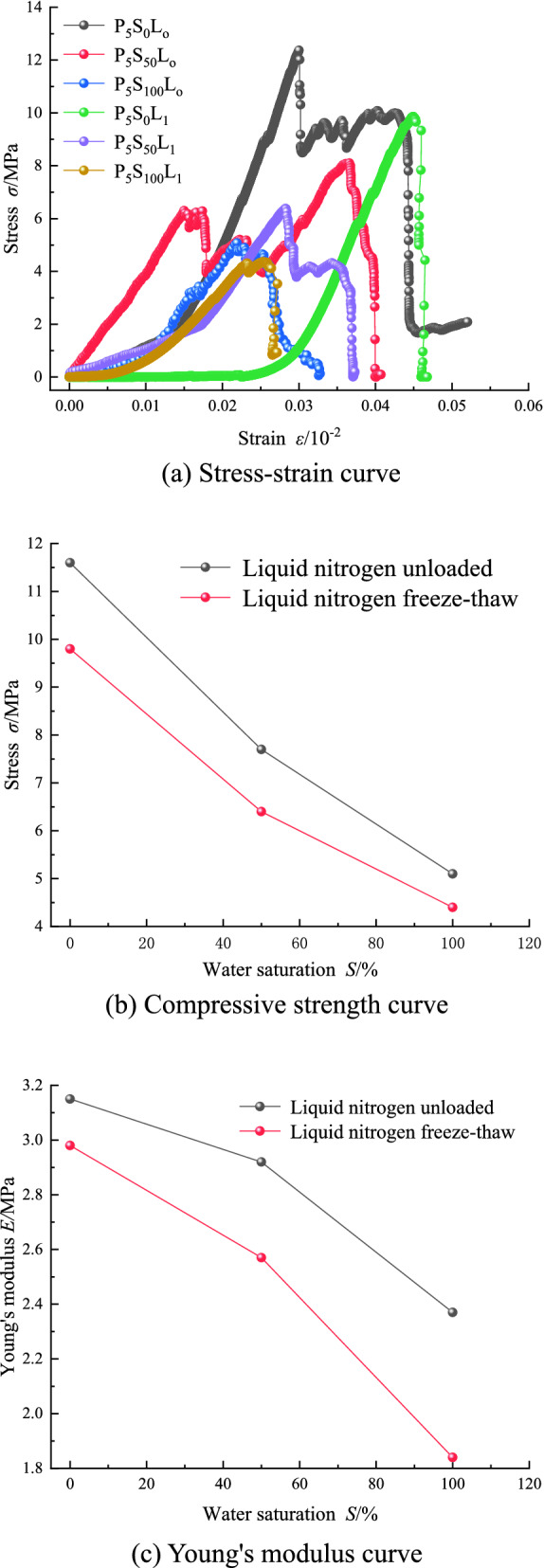
Test result curve of coal sample strength damage analysis.

After the liquid nitrogen freeze–thaw test, the confining pressure was withdrawn, and the MH-25 universal testing machine was used to carry out the uniaxial compression test of the coal sample, and the loading rate was 0.1 mm/min. Draw the stress–strain curve, the compressive strength curve, and Young's modulus curve, as shown in Fig. 6a–c.

It can be seen from Fig. 6a–c that in the comparative test ***A***, when liquid nitrogen is unloaded, with the increase of water saturation, the damage to the coal sample joint structure is aggravated, and the compressive strength and Young's modulus gradually decrease. The compressive strengths of 0%, 50%, and 100% water saturation are 11.6 MPa, 7.7 MPa, and 5.1 MPa, respectively, and Young's moduli are 3.15GPa, 2.92GPa, and 2.37GPa, respectively. Under the condition of freezing and thawing of liquid nitrogen in Comparative Experiment ***B***, with the increase of water saturation, the damage degree of coal sample joint structure gradually increases, and the compressive strength also decreases obviously. The compressive strengths of 0%, 50%, and 100% water saturation are 9.8 MPa, 6.4 MPa, and 4.4 MPa, respectively, and Young's moduli are 2.98 GPa, 2.57 GPa, and 1.84 GPa, respectively. Compared with the effect of liquid nitrogen unloaded, the compressive strength has a greater decrease after liquid nitrogen freezing and thawing. According to the analysis of Griffith's theory^37–39^, it is believed that the deterioration of compressive strength of coal samples is caused by further damage to the fracture structure of the rock mass. Liquid nitrogen freezing and thawing cause damage to the joint structure of the coal sample. With the increase of the water saturation of the coal sample, after a single cycle of freezing and thawing, the damage to the overall joint and pore structure of the coal sample intensifies, and the primary joints on the surface of the coal sample expand and generate new joints. As a result, the skeleton of the coal matrix that plays a supporting role is partially damaged, the compressive strength of the coal sample is reduced, Young's modulus becomes smaller, and the overall damage degree of the coal sample is aggravated. The stress–strain relationship curve obtained in this paper is consistent with the theoretical deduction of Zhou et al.^7–10^, which further verifies the correctness of the research results in this paper.

### Analysis of the expansion force of coal sample joint ice-wedge under the action of liquid nitrogen freezing and thawing

#### Assumptions of the physical and mechanical model of freeze–thaw damage

**Figure 7:**
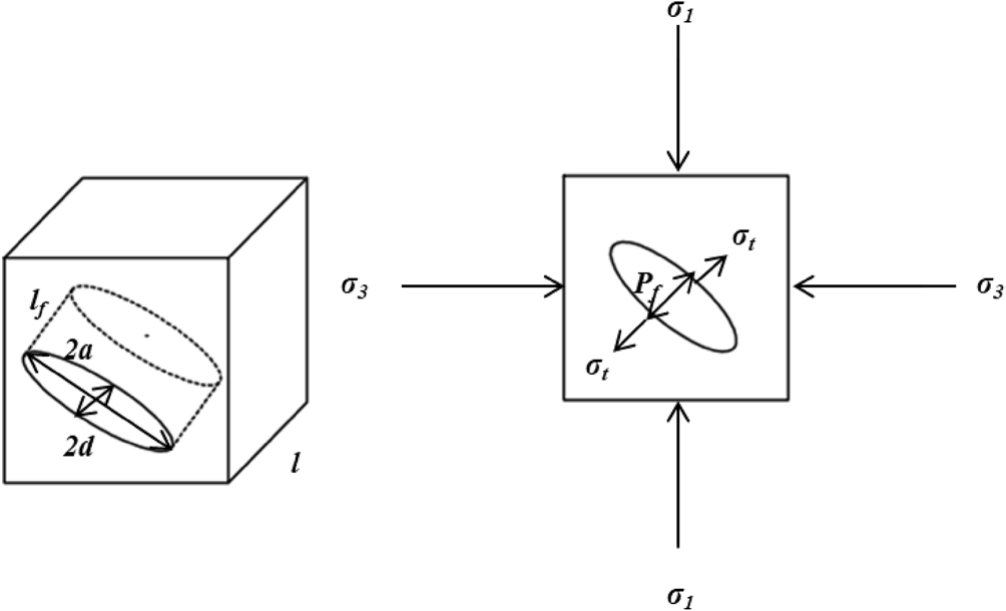
Freeze–thaw damage physical and mechanical model.

The water in the coal sample joint freezes into ice under the action of liquid nitrogen and forms an ice wedge, which is embedded in the coal sample joint and causes compressive stress on the joint wall and the joint tip, that is, the ice-wedge expansion stress. Under the action of liquid nitrogen freezing and thawing, the joint structure of the water-bearing coal sample is mainly affected by the temperature stress $\upsigma _{t}$, the ice-wedge expansion stress $P_{f}$, the confining pressure $\upsigma _{3}$, and the vertical compressive stress $\upsigma _{1}$. To explore the ice-wedge expansion stress in the joint, the following assumptions are made with full consideration of the actual working conditions:

1. In the test, $\upsigma _{1}$ only plays the role of limiting the displacement, so it can be ignored.
2. The coal sample is impermeable, the water saturation in the joint is 100%, the volume and mass of water remain unchanged during the extrusion process, and the process of water mass migration conforms to the cubic law.
3. Ice and coal matrix belong to isotropic elastic medium material.
4. Due to the unfrozen water film attached to the ice surface^40^, it is considered that there is no friction between the ice and the joint will of the coal sample, and the water pressure is evenly distributed along the joint direction during the volume expansion process.
5. The cross-section of the joint remains elliptical^41,42^, and the physical and mechanical model of freeze–thaw damage is shown in Fig. 7.

#### Moisture migration equation

The transformation of liquid water into solid ice in the joint structure of coal samples is not instantaneous but accompanied by a change process. During the phase transition, the water that first contacts the liquid nitrogen forms ice expands its volume, and increases its water pressure, causing expansion force. The water mass (water mass including liquid-phase water, ice-water mixture, and initial ice) in the drive coal sample joint is extruded outward along the joint or pore-crack channel, and the amount of extruded water mass is affected by temperature and phase transition rate. At the same time, the amount of water body also affects the expansion stress, which can be deduced by using the expansion pressure formula^43–46^.

In the process of ice-wedge expansion, the increase of joint water pressure produces a water pressure gradient that causes joint water to migrate outward, ignoring the effects of free water gravitational potential and capillary potential. The flow rate of water bodies in coal sample joints, pores, and fissures can be expressed as

3 $$ Q = 2a\int\limits_{T^{\prime}} {q{\text{d}}t} = \int\limits_{T^{\prime}} { - \gamma } \frac{{4a\lambda^{3} d^{3} }}{3\mu }\frac{{\partial P_{{\text{f}}} }}{\partial l}{\text{d}}t $$

4 $$ q = \left[ {\gamma \left( {2\lambda d} \right)^{3} /\left( {12\mu } \right)} \right](\partial P_{f} /\partial l) $$

In Eqs. (3) and (4), $$T^{\prime } $$ is the time, min; $$Q$$ is the volume of the water body extruded during the time $$T^{\prime }$$, ml; $$q$$ is the flow rate per unit width of the joint in unit time along the joint direction, which conforms to the cubic law; $$\upmu $$ is the dynamic viscosity coefficient; $$P_{f}$$ is the ice-wedge expansion stress during the freeze–thaw process (the same as the water pressure stress during the freeze–thaw process), MPa; $$\upgamma $$ is the joint water weight, g/cm^3^; $$\uplambda $$ is the correction coefficient related to the joint width, etc. If the equivalent hydraulic width correction is adopted, it is preferable: $$\lambda = \sqrt[3]{3\pi /16}$$$${3\pi /16}$$; $$a$$ is the distance of the long axis of the ellipse; $$d$$ is the distance of the short axis of the ellipse; $$l$$ is the distance of the water migration direction of the joint.

#### Volume expansion coupling equation

If the length of the freeze–thaw action section in the coal sample joint is $l_{f}$, and $l_{f} < l$, in the freeze–thaw action section, the volume expansion of the water–ice phase transition under unconstrained conditions is

5 $$ \Delta V_{{\text{f}}}^{^{\prime}} = \beta \left( {V_{{\text{f}}}^{0} - Q} \right)u^{t} $$

In Eq. (5), $\beta$ is the volume expansion coefficient of water–ice phase transition under the condition of a free state, which is a dimensionless constant; $u^{t}$ is the freezing rate of freezing and thawing at temperature *t*, %; $V_{{\text{f}}}^{0}$ is the joint volume before freezing and thawing.

The joint volume before freezing and thawing is

6 $$ V_{{\text{f}}}^{0} = {\uppi }adl_{{\text{f}}} $$

Considering the confinement effect of the joint wall on the ice, under the action of the uniform expansion stress $P_{f}$, the ice volume in the joint will be squeezed and deformed relative to the unconstrained condition. From the elastic mechanics, it can be known that the volume strain under the plane strain is:

7 $$ \varepsilon_{{\text{i}}}^{V} = \frac{{3\left( {1 - 2\upsilon_{i}^{t} } \right)}}{{E_{i}^{t} }}P_{{\text{f}}} $$

In Eq. (7), $E_{i}^{t}$ is Young's modulus of ice under the condition of temperature *t,* GPa; $\upsilon_{i}^{t}$ is the Poisson's ratio of ice.

The actual volume of ice in the joint under the constraints of the joint wall is

8 $$ V_{{\text{f}}} = \left( {V_{{\text{f}}}^{0} - Q + \Delta V_{{\text{f}}}^{^{\prime}} } \right)\left( {1 - \varepsilon_{i}^{V} } \right) $$

After 1 cycle of freeze–thaw action, the volume of the joints is

9 $$ V_{{_{{\text{f}}} }}^{1} = {\uppi }\left( {a + \Delta a} \right)\left( {d + \Delta d} \right)l_{{\text{f}}} $$

In Eq. (9), $V_{{\text{f}}}^{1}$ is the joint volume of the coal sample after one cycle of freezing and thawing, μm^3^; $\Delta a$ and $\Delta d$ are the changes in the long and short axis dimensions of the coal sample joint cross-section under the action of the ice-wedge expansion stress, respectively, μm.

After freezing and thawing, the ice in the joint will fill the joint section of length $l_{f}$, and the volume of ice in the coal sample joint is equal to the joint volume, so there is an expansion coupling relationship.

10 $$ V_{{\text{f}}} = V_{{_{{\text{f}}} }}^{1} $$

**Figure 8:**
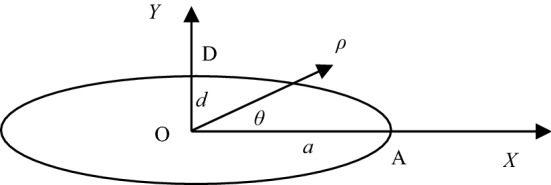
Schematic diagram of the oval joint plan.

After freezing and thawing, the volume of liquid water into solid ice has been obtained, and the key is to continue to solve the geometric relative expansion of coal sample joints. For plane elliptical joints under uniformly distributed internal pressure, the displacement of the joint's long and short axes can be calculated by using elastic mechanics combined with complex function theory, as shown in points A and D in Fig. 8.

The calculated displacement at the center point *A* of the long axis is:

11 $$ u_{r}^{{\text{A}}} = \frac{{P_{{\text{f}}} R}}{{2G_{{\text{S}}}^{t} }}\left( {1 - \frac{{3 - \upsilon_{{\text{S}}}^{t} }}{{1 + \upsilon_{{\text{S}}}^{t} }}m} \right) $$

The displacement at the center point D of the short axis is:

12 $$ u_{r}^{{\text{D}}} = \frac{{P_{{\text{f}}} R}}{{2G_{{\text{S}}}^{t} }}\left( {\frac{{3 - \upsilon_{{\text{S}}}^{t} }}{{1 + \upsilon_{{\text{S}}}^{t} }}m + 1} \right) $$

In Eqs. (11) and (12), $G_{{\text{S}}}^{t} = {{E_{{\text{S}}}^{t} } \mathord{\left/ {\vphantom {{E_{{\text{S}}}^{t} } {{{\left( {1 - \upsilon_{{\text{S}}}^{t} } \right)} \mathord{\left/ {\vphantom {{\left( {1 - \upsilon_{{\text{S}}}^{t} } \right)} 2}} \right. \kern-\nulldelimiterspace} 2}}}} \right. \kern-\nulldelimiterspace} {{{\left( {1 - \upsilon_{{\text{S}}}^{t} } \right)} \mathord{\left/ {\vphantom {{\left( {1 - \upsilon_{{\text{S}}}^{t} } \right)} 2}} \right. \kern-\nulldelimiterspace} 2}}}$ is the shear modulus of the coal sample under the condition of temperature *t*, GPa; $E_{{\text{S}}}^{t}$ is Young's modulus of the coal sample under the condition of temperature *t*, GPa; $\upsilon_{{\text{S}}}^{t}$ is the Poisson's ratio of the coal sample at temperature *t*. Among them,$m = {{\left( {a - d} \right)} \mathord{\left/ {\vphantom {{\left( {a - d} \right)} {\left( {a + d} \right)}}} \right. \kern-\nulldelimiterspace} {\left( {a + d} \right)}}$, $R = {{\left( {a + d} \right)} \mathord{\left/ {\vphantom {{\left( {a + d} \right)} 2}} \right. \kern-\nulldelimiterspace} 2}$, when $a > > d$, ignoring the joint width, there is $m = 1$, $R = {a \mathord{\left/ {\vphantom {a 2}} \right. \kern-\nulldelimiterspace} 2}$.

Therefore, the joint width variation at the center of the short axis and the long axis is simplified as

13 $$ \Delta d = u_{r}^{D} = \frac{{P_{{\text{f}}} }}{{G_{{\text{S}}}^{t} }}\frac{a}{{1 + \upsilon_{{\text{S}}}^{t} }} $$

14 $$ \Delta a = u_{r}^{{\text{A}}} = - \frac{{P_{{\text{f}}} }}{{2G_{{\text{S}}}^{t} }}\frac{{a\left( {1 - \upsilon_{{\text{S}}}^{t} } \right)}}{{1 + \upsilon_{{\text{S}}}^{t} }} $$

When $a \gg d$ the length change of the approximately straight ellipse joint in the *x*-direction $\left| {\Delta a} \right| = \left| {\Delta d} \right|{{\left( {1 - \upsilon_{{\text{S}}}^{t} } \right)} \mathord{\left/ {\vphantom {{\left( {1 - \upsilon_{{\text{S}}}^{t} } \right)} 2}} \right. \kern-\nulldelimiterspace} 2}$ is a minimum value, it can be considered $\Delta a \times \Delta d \approx 0$ that the Eq. (9) can be simplified as

15 $$ V_{{_{{\text{f}}} }}^{1} = {\uppi }\left( {ad + a\Delta d + \Delta ad} \right)l_{{\text{f}}} $$

Substituting Eqs. (13) and (14) into the above Eqs. (15), we have

16 $$ V_{{_{{\text{f}}} }}^{1} = {\uppi }a\left( {d + \frac{{P_{{\text{f}}} }}{{G_{{\text{S}}}^{t} }}\frac{a}{{1 + \upsilon_{{\text{S}}}^{t} }} - \frac{{P_{{\text{f}}} }}{{2G_{{\text{S}}}^{t} }}\frac{{1 - \upsilon_{{\text{S}}}^{t} }}{{1 + \upsilon_{{\text{S}}}^{t} }}d} \right)l_{{\text{f}}} $$

Substituting Eqs. (5), (7), (8), and (16) into Eq. (10), the expansion coupling relationship can be obtained:

17 $$ \left[ {V_{{_{{\text{f}}} }}^{0} - Q + \beta \left( {V_{{_{{\text{f}}} }}^{0} - Q} \right)u^{t} } \right]\left[ {1 - \frac{{3\left( {1 - 2\upsilon_{{\text{S}}}^{t} } \right)}}{{E_{{\text{S}}}^{t} }}P_{{\text{f}}} } \right] = {\uppi }a\left( {d + \frac{{P_{{\text{f}}} }}{{G_{{\text{S}}}^{t} }}\frac{a}{{1 + \upsilon_{{\text{S}}}^{t} }} - \frac{{P_{{\text{f}}} }}{{2G_{{\text{S}}}^{{\text{t}}} }}\frac{{1 - \upsilon_{{\text{S}}}^{t} }}{{1 + \upsilon_{{\text{S}}}^{t} }}d} \right)l_{{\text{f}}} $$

Assuming that the proportional coefficient between the outflow water and the original volume water is $\xi$, we have

18 $$ Q = \xi V_{{\text{f}}}^{0} $$

In Eq. (18), $V_{{\text{f}}}^{0}$ is the volume before freezing and thawing.

Substitute Eqs. (6) and (18) into Eq. (17) to obtain the relationship between expansion stress, mechanical parameters of ice in coal samples, and geometric parameters of joints

19 $$ P_{{\text{f}}} = \frac{{k_{i} - 1}}{{\frac{{k_{i} }}{{K_{i}^{t} }} + \left( {\frac{a}{d} - \frac{{1 - \upsilon_{{\text{S}}}^{t} }}{2}} \right)\frac{1}{{G_{{\text{S}}}^{t} \left( {1 + \upsilon_{{\text{S}}}^{t} } \right)}}}} $$

In Eq. (19), $k_{i} = \left( {1 + \beta u^{t} } \right)\left( {1 - \xi } \right)$ is the volume expansion coefficient of the water in the joint after the water body is extruded; $K_{{_{i} }}^{t} = E_{{_{{\text{i}}} }}^{t} /\left( {1 - 2\upsilon_{i}^{t} } \right)/3$ is the bulk modulus of the ice in the joint.

Using Eq. (19), the ice-wedge expansion stress caused by freezing and thawing of water in coal sample joints with a water saturation of 100% under the action of liquid nitrogen can be calculated, where $\rho$ is the polar diameter, $\uptheta$ is the polar angle. It can be seen from fracture mechanics that under the action of the expansion stress $P_{{\text{f}}}$, the theoretical solution of the stress field at the tip of the joint inside the coal sample can be expressed in the polar coordinate system as

20 $$ \sigma \left( {\rho ,0} \right) = P_{{\text{f}}} $$

In Eq. (20), the ice-wedge expansion stress $P_{{\text{f}}}$ is the tensile stress, MPa.

**Figure 9:**
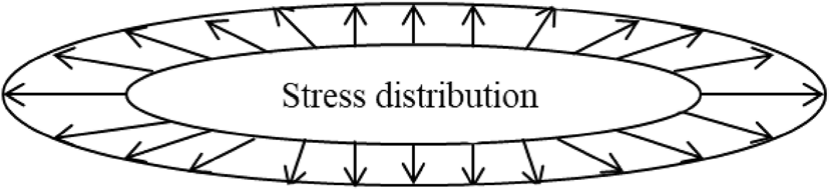
Schematic diagram of stress distribution of ellipsoid coal sample joint model.

Figure 9 shows the effect of ice-wedge expansion stress on elliptical coal sample joints.

It can be seen that there is a tensile stress perpendicular to the long axis at the tip of the coal sample joint, and its value is the same as the expansion stress. Therefore, the joint damage criterion of coal samples under the action of liquid nitrogen freezing and thawing can be established.

21 $$ \sigma_{1t} \le P_{{\text{f}}} $$

In Eq. (21), $\sigma_{1t}$ is the tensile strength of the coal sample, MPa.

Under the action of ice-wedge expansion stress, the tip of the coal sample joint structure is first damaged by stress. The tensile strength of long flame coal in the Fuxin Basin is about 0.52 MPa. After freezing and thawing of liquid nitrogen, the liquid phase water in the coal sample joint turns into solid-phase ice and squeezes the joint wall. The ice wedge expansion stress generated after a single cycle of freezing and thawing exceeds the tensile strength of the coal sample, which will cause local damage to the joint tip. With the increase of joint length and width, the capacity of ice-wedge expansion stress gradually weakens, but the joint still expands gradually, gathers and penetrates, and at the same time increases the permeability, the coal sample is destroyed.

## Conclusion

Ice wedge expansion stress and joint expansion of coal-rock joints under the action of liquid nitrogen at low temperatures have always been the key scientific issues in the study of freezing damage of rock engineering in cold and arid regions. However, due to the multi-disciplinarity involved, there are many difficulties to be studied, and the research on liquid nitrogen freezing and thawing of coal is far from mature. Based on previous research, this paper conducts a preliminary study on the damage law and mechanism of a coal-rock joint structure under the action of liquid nitrogen at low temperatures. The main conclusions are as follows:

1. The water in the coal sample joints forms ice wedges under the action of liquid nitrogen freezing and thawing, and the expansion stress of the ice wedges is greater than the tensile strength of the coal samples, which causes damage and expansion of the water-bearing joints and pore-cracks in the coal matrix. With the increase of water saturation, the damage degree gradually intensified, and Young's modulus of coal samples decreased.
2. Considering the characteristics of water migration and water–ice phase transition in saturated joints, a physical and mechanical model of freeze–thaw damage is established, and the solution formula and joint damage criterion are proposed. It is found that the tip of the coal sample joint is more prone to stress concentration and damage, and the damage to the coal sample joint is damage expansion along the joint direction.
3. Under the combined action of temperature stress, confining pressure, and ice-wedge expansion stress, the ice-wedge expansion stress generated by water in liquid nitrogen freeze–thaw joints is the most critical stress form for the structural damage of coal sample joints. The test results have a good correspondence with the theoretical model, and the damage law is consistent, which shows that it is feasible to use the freeze–thaw damage physical–mechanical model to reveal the damage law of coal samples.

## Data Availability

The data used to support the findings of this study are available from the corresponding author upon request.
